# The Interaction between Four Polymorphisms and Haplotype of *ABCB1*, the Risk of Non-Small Cell Lung Cancer, and the Disease Phenotype

**DOI:** 10.1155/2023/7925378

**Published:** 2023-01-24

**Authors:** Agnieszka Jeleń, Marta Żebrowska-Nawrocka, Aleksandra Sałagacka-Kubiak, Izabela Zawadzka, Mariusz Łochowski, Ewa Balcerczak

**Affiliations:** ^1^Laboratory of Molecular Diagnostics and Pharmacogenomics, Department of Pharmaceutical Biochemistry and Molecular Diagnostics, Medical University of Lodz, Lodz, Poland; ^2^Department of Thoracic Surgery, Memorial Copernicus Hospital, Medical University of Lodz, Lodz, Poland

## Abstract

P-glycoprotein, product of the *ABCB1* (ATP binding cassette subfamily B member 1) gene, has been reported to play an important role in multiple drug resistance during cancer therapy. However, its influence on non-small cell lung cancer (NSCLC) risk has not been clearly defined. The aim of the present study was to examine the association between clinicopathological factors and SNPs T-129C, C1236T, G2677T/A, and C3435T, as well as its haplotype, and to investigate the role of ABCB1 polymorphisms in NSCLC development. The study included 80 patients who suffered from NSCLC and underwent surgery to remove the tumour and 96 healthy controls. The tissues were genotyped by PCR-RFLP and sequencing methods, and the haplotype frequencies in both groups were estimated. The SNP C3435T was identified as a NSCLC risk factor. The presence of mutated allelic variant T (*p*=0.0103) or homozygote TT (*p*=0.0099) was observed significantly more often in cancer patients than in healthy controls. The two groups also demonstrated a highly significant difference in common haplotype frequency (*p*=0.01). The T_−129_-T_1236_-T_2677_-T_3435_ haplotype was found to be most closely associated with NSCLC risk. Although the investigated polymorphisms were not related to demographic features, clinicopathological lung tumour characteristics, or blood morphology indices, marginally significant correlations were found with some variables: C1236T with age of disease onset (*p*=0.0410); C3435T with smoking status (*p*=0.0561). As the findings indicate, lung cancer and control groups demonstrate significantly different patterns of −129/1236/2677/3435 haplotype distribution; T-T-T-T haplotype contributes to NSCLC susceptibility, and this effect is probably mainly dependent on C3435T. So far, similar studies were published in other populations.

## 1. Introduction

Lung cancer is one of the most common cancer types in terms of both incidence and mortality [1]. Worldwide, more than 1.8 million lung cancer deaths occurred in 2020 [2]. In 2019, lung cancer was the second most prevalent newly diagnosed cancer and still the dominant cause of cancer death in both men and women, according to the Polish National Cancer Registry [3].

Lifestyle risk factors play key roles in lung cancer development. While the genome determines susceptibility to cancer or provides protection against the initiation and/or promotion of carcinogenesis, morbidity and mortality rates are usually correlated with exposure to tobacco smoking and outdoor/indoor air pollution [1, 2]. In addition, exposure of particular cells to toxic metabolites, drugs, and over-the-counter medications influences the biological mechanisms contributing to cancer cell development and growth.

One of the most important cellular transporters determining the concentration of organic cations, carbohydrates, amino acids, proteins, polysaccharides, certain cytokines, and antibiotics in the different parts of the body is P-glycoprotein (P-gp), encoded by the *ABCB1* gene. The principal role of P-gp is to protect against xenobiotics entering the human body from the environment/diet; as such, the protein is typically localised at sites of exposure to foreign compounds [4, 5]. Tumour cells exploit the protective function of P-gp, increasing its level in response to treatment with cytostatic drugs. Moreover, while *ABCB1* mRNA level is typically found at an intermediate level in normal lung tissue, and this value is downregulated in lung tumour cells [6]. The decrease of P-gp may disturb the balance between the extracellular and intracellular environments and exacerbate the changes in the concentrations of compounds which may lead to cancer cell formation. However, as previously shown, patients with lung tumour present a similar level of *ABCB1* expression in cancer tissue and blood cells [7]. Hence, it appears that diverse exogenous and endogenous compounds, which have a strong influence on P-gp function, may influence the normal, i.e., genetically-determined, level of protein activity. In addition, the presence of highly frequent polymorphisms in the *ABCB1* sequence may contribute to cancer development and significantly determine the disease phenotype.

The presented study tests the hypothesis that variations (T-129C, C1236T, G2677T/A, and C3435T) in the *ABCB1* gene, which forms a biological barrier against toxins/xenobiotics, may influence the risk of lung cancer and may be a prognostic marker in patients with non-small cell lung cancer (NSCLC). Candidate polymorphisms were selected on the basis of prior literature reporting a significant effect of these SNPs on the ABCB1 protein and/or their correlation with the clinical course of many diseases.

## 2. Materials and Methods

The whole blood specimens from patients with non-small cell lung cancer were obtained from Nicolaus Copernicus Regional Specialist Hospital in Lodz (Lodz, Poland). Materials were collected between March 2016 and December 2020. All experiments performed in this study were in accordance with the principles of the Declaration of Helsinki and approved by the Ethical Committee of the Medical University of Lodz (No RNN/87/16/KE). All the included individuals gave informed consent.

### 2.1. Study Group

Eighty patients with NSCLC (age at diagnosis between 32 and 82 years; median 68 years old) undergoing surgical resection were enrolled in this study. Inclusion criteria for this group are histopathological confirmation indicating NSCLC and its surgical treatment. The diagnosis of small cell lung cancer or carcinoid recognized after histologic examination were the exclusion criteria. Information concerning age, gender, and smoking status was collected from each patient. At the time of diagnosis, subjects were also clinically staged according to the TNM system. Following analysis of the surgical specimen, the histological type and tumour grade were identified. The profile of the investigated group is given in Table 1. Seventeen of the study subjects were treated after surgery with adjuvant chemotherapy consisting of combined cisplatin E and vinorelbine or carboplatin and gemcitabine.

### 2.2. Control Group

A control group was formed from 96 voluntary blood donors from the regional blood bank who were residing in central Poland. The samples were only tested for T-129C; they had previously been tested for three *ABCB1* polymorphisms (C1236T, G2677T/A, and C3435T) in the Laboratory of Molecular Diagnostics and Pharmacogenomics [8].

## 3. Methods

### 3.1. DNA Isolation

DNA from peripheral blood was isolated according to “Blood Mini” protocol (A&A Biotechnology, Poland). The concentration and purity of DNA samples were assessed using a UV/VIS nanospectrophotometer (Implen). Until analysis, the DNA samples were stored at −20°C.

### 3.2. Polymerase Chain Reaction (PCR)

For studied polymorphisms, the PCR reactions were conducted according to “2x DreamTaq Green PCR Master Mix” (Thermo Fisher Scientific) protocol in 25 *µ*l volumes of PCR mixture: 12.5 *µ*l of 2x DreamTaq Green PCR Master Mix, 0.5 *µ*l of 10 *µ*M forward and reverse primers specific for each of the studied SNPs, and 50 ng of DNA. The mixture was made up to 25 *µ*l with distilled, sterilized water. Each experiment included a negative control, i.e., without DNA. Products of the PCR reactions were assessed by electrophoresis in 2% agarose gel. The reaction products for the SNPs at positions T-129C, C1236T, G2677T/A, and C3435T were 258, 370, 262, and 208 bp in length, respectively.

### 3.3. Restriction Fragment Length Polymorphism (RFLP)

The polymorphisms T-129C and 3435 were genotyped by RFLP. Briefly, the PCR products of SNP T-129C were digested by *MspA1I*. The reaction mixture consists of 2 *µ*l of 10x buffer, 0.2 *µ*l restriction enzyme 10 U/*µ*l, 1.8 *µ*l of distilled water, and 16 *µ*l of PCR product. Restriction was performed at 37°C for 16 hours. Genotypes for SNP T-129C were identified by electrophoresis after digestion by *MspA1I* (one band of 258 bp for genotype TT; two bands of 226 and 32 bp for genotype CC; three bands of 258, 226, and 32 bp for genotype CT). The RFLP procedure used for genotyping the polymorphism at position 3435 was performed according to Zawadzka et al. [7].

### 3.4. Sequencing

Genotyping for polymorphism 1236 was performed by Sanger sequencing. The sequencing-PCR reaction (SeqPCR) was conducted according to the “Thermo Sequenase Dye Primer Manual Cycle Sequencing Kit” protocol (Thermo Fisher Scientific). The reaction mixture for SeqPCR was composed of the following: 2.2 *µ*l of concentrate reaction buffer, 1 *µ*l of Thermo Sequenase DNA Polymerase (20 U/*μ*l), 1 *µ*l of 10 *µ*M primer (labeled by IRD800 at 5′end), 1 *µ*l of each dNTP/ddNTP mixture, and 2 *µ*l of amplified DNA. The mixture was made up to a final volume of 20 *µ*L with distilled water. After sequencing, PCR amplification loading buffer was used. The SeqPCR products were separated into 40% polyacrylamide gels. Sequencing was performed with a LI-COR® DNA Analyzer 4300 automated sequencer (Supplement Figure 1).

Sequencing for polymorphism 2677 was performed by the GENOMED Company (Warsaw, Poland). In this case, the sequencing reactions were performed using the BigDye® Terminator v3.1 kit from Applied Biosystems (Thermo Fisher Scientific). The sequencing reaction products were separated on a 3730x1 DNA Analyzer capillary sequencer (Supplement Figure 2).

All PCR and SeqPCR primer sequences have been published previously [7, 9, 10].

### 3.5. Statistics

A chi-square statistic with or without Yates correction, odds ratios (OR), and 95% confidence intervals (95% CI) were used to evaluate the associations between the investigated SNPs of *ABCB1* and NSCLC risk. The same statistical test was also used to check whether the study group is in equilibrium according to the Hardy–Weinberg principle. The type of statistical test used for analyzing the other quantitative and qualitative clinical data is given in the corresponding supplementary tables (Supplement Tables A1–A4 and B1–B4).

Linkage disequilibrium (LD) between particular SNPs was estimated based on the *r*-squared statistic using EMLD software. The *D*′ and *r*^2^ parameters were estimated for pair-wise combinations of investigated SNPs. Haplotype frequencies from genotyping data were estimated using PHASE, version 2.1 [11, 12].

In all conducted tests, a value of *p* < 0.05 was considered significant.

## 4. Results

All NSCLC cases were successfully genotyped and included in the analysis. Most were male, and over half were current smokers. The most common form was squamous cell carcinoma. Almost half of the cancer cases were identified in TNM stages II or III, and most had moderately well-differentiated tumour cells (G2). More detailed demographic and clinicopathological characteristics of this group are presented in Table 1.

### 4.1. Association of Genotype and Haplotype with Lung Cancer Risk

In this group, T-129C, C1236T, G2677T/A, and C3435T were all found to be in line with the Hardy–Weinberg equilibrium (*p* > 0.05). The genotyping results were compared to the control values. In both the study and control groups, the −129C allele was identified in four subjects, none of whom were homozygous (Table 2).

T-129C did not appear to be related to cancer risk. C1236T was associated with an NSCLC risk, with the 1236 CT genotype demonstrating a lower risk of NSCLC than the CC genotype (*p*=0.0480). In contrast, the difference between the frequencies of T and C alleles between the study and control groups was not significant (*p*=0.7535). For G2677T/A, the frequency of the rare mutant allele A was 5%. Because the study group contained only one TA and three GA carriers, these subjects were included in the mutated homozygous (TT) and heterozygous (GT) groups, respectively, for data analysis. For this SNP, significant differences in neither genotype nor allele frequencies between the study and control groups were stated (*p*=0.1116 and *p*=0.2104). Finally, for C3435T, significant differences in genotype frequencies and allele distribution were noted between cases and controls. The lung cancer patients were more likely to harbour a TT genotype, and this was associated with a more than threefold higher risk of NSCLC (OR 3.27; 95% CI 1.33–8.06; *p*=0.0099) than that of CC genotype. Accordingly, the T allele was more frequent in cancer patients than in healthy controls and connected with elevated risk of NSCLC (OR 1.75; 95% CI 1.14–2.67; *p*=0.0103). Table 2 summarizes the results of four *ABCB1* SNPs genotyping in patients with NSCLC and shows also genotypes/alleles distribution in healthy controls.

**Figure 1:**
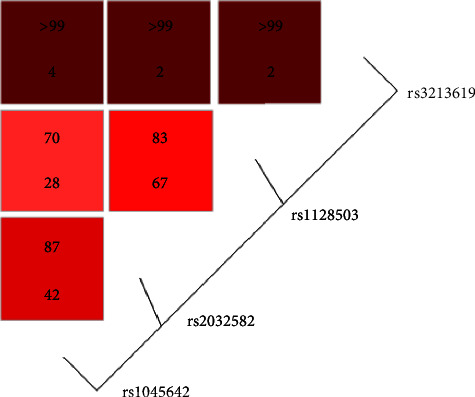
LD between *ABCB1* polymorphisms. The numbers within the squares represent *D*' (upper) and *r*^2^ (lower) value as percentages.

The three most extensively studied SNPs of *ABCB1* are known to be in strong linkage disequilibrium. Moreover, polymorphisms present in the gene regulatory region directly influence the expression of the gene and may form important parts of this haplotype. Our present findings confirm a disequilibrium between every pair of the four investigated *ABCB1* SNPs: the *D*′ values range from 0.999 to 0.700, with the highest disequilibrium observed between T-129C and the other SNPs. The *r*^2^ values ranged from 0.667 (value between C1236T and G2677T/A) to 0.023 (value between T-129C and C1236T or G2677T/A). T-129C is much rarer than the others, as indicated by the high *D*′ parameter value together with a low *r*^2^ value (Figure 1).

To determine the effect of haplotype on NSCLC risk, the haplotype frequencies in lung cancer patients and healthy individuals were estimated, and the two groups were compared. The most common haplotypes in the study group were T_−129_-T_1236_-T_2677_-T_3435_ (0.3730) haplotype, followed by T_−129_-C_1236_-G_2677_-C_3435_ (0.3178). In the control group, the most common was T_−129_-C_1236_-G_2677_-C_3435_ (0.2087). Both T_−129_-C_1236_-T_2677_-T_3435_ and T_−129_-T_1236_-G_2677_-C_3435_ were observed quite frequently in the control group (0.1300 and 0.1496, respectively). All these estimated haplotypes were found to differ significantly in frequency between patients and controls (*p*=0.01); however, the T_−129_-T_1236_-T_2677_-T_3435_ haplotype was not only significantly different in frequency between the two groups, but it was also the most closely associated with NSCLC risk.

### 4.2. Association with Clinicopathological Features of Disease

We hypothesized that SNPs, which show significant association with risk of NSCLC, may also correlate with patient demographics or the pathological features of the tumour. To verify this hypothesis, the clinicopathological data of the lung cancer patients (i.e., age, sex, cigarette smoking status, cancer type, overall stage, and differentiation grade) were compared with the single SNP genotyping results.

No statistically significant correlation was found between the analyzed clinical parameters and the investigated *ABCB1* gene polymorphisms (Supplement Tables A1–A4 and B1–B4). However, C1236T was correlated with age of disease onset and C3435T with smoking status. In addition, patients with heterozygous C1236T (CT) tended to be at greater age when the lung cancer was diagnosed and surgically resected compared to homozygous patients (TT) (*p*=0.0410). This observation was not reflected in the analysis of allelic variants of C1236T (Supplement Table B2). Also, nonsmokers with lung cancer were more likely to present at least one 3435 C allele than the tobacco smoking patients (*p*=0.0200). Comparisons of the frequencies of the C3435T genotypes between patients stratified according to smoking status were of only borderline significance (Supplement Table A4).

### 4.3. Association with Blood Morphology Indices

Inflammation plays an important role in the initiation, promotion, and progression of cancer. Therefore, blood morphology indices could be prognostic factors in lung cancer patients. The final part of the analysis examined the potential association between the genotypes/allelic variants of *ABCB1* SNPs and selected blood indices: red blood cell count, white blood cell count (and its particular components such as neutrophils, lymphocytes, and monocytes), platelet, hematocrit, and immunoglobulin level, and platelet-to-lymphocyte (PLR), neutrophil-to-lymphocyte (NLR), and lymphocyte-to-monocyte (LMR) ratio. No association was found between the investigated parameters. A detailed list of results obtained for particular SNPs is shown in the supplementary material (Supplement Tables B1–B4).

## 5. Discussion

The protein encoded by the *ABCB1* gene plays a key role in the defence of respiratory tract cells against xenobiotics, as demonstrated by its presence, *inter alia*, in the bronchial epithelium, mucinous glands, alveolar cells, endothelium, and normal nasal respiratory mucosa [13]. Additionally, *ABCB1* gene expression is reduced in lung cancer tissue taken during surgery compared to normal cells [6]. It has been suggested that some allelic variants present in this gene may induce interindividual differences in P-gp activity, by influencing mRNA level, protein formation, cellular concentrations, or substrate specificity [14–17]. This *ABCB1* genetic variability can support the process of carcinogenesis at different stages, acting as a risk factor or modifying the disease phenotype.

Despite the high number of polymorphisms identified in the *ABCB1* gene and the fact that the most frequent allelic variants have been studied in various diseases, the impact of particular SNPs on lung cancer development remains tentative. In response to the limited number of studies addressing the influence of *ABCB1* on NSCLC risk and course of disease, the present study examined the relationships between four selected SNPs and their haplotype with NSCLC susceptibility, patient demographic characteristics, tumour pathomorphological features, and blood morphology. As a result, this study is the first such comprehensive analysis of these SNPs, lung cancer risk, and disease phenotype in a Polish population.

Our studies found the frequencies of *ABCB1* −129C, 1236T, 2677T/A, and 3435T alleles to be 5.0%, 62.5%, 67.9%, and 86.3%, respectively. The allele frequency distribution did not deviate significantly from data published for a similar Caucasian population (https://www.ncbi.nlm.nih.gov/snp/) for lung cancer [18, 19] or other malignant tumours [20, 21]. Our data indicates that for SNP 3435, the TT genotype and the T allele were more common in lung cancer patients than in healthy subjects, and the presence of this genotype (TT) increased the risk of lung cancer by more than threefold. Our data confirm those of Subhani et al. (2015) in a South Indian lung cancer population or Sheng et al. (2012) in Caucasian and Asian patients with hematologic malignancies, breast cancer, and renal cancer [22, 23]. However, the latter meta-analysis based on two studies by Sheng et al. did not confirm any association between lung cancer incidence and C3435T genotype in the Caucasians. A similar analysis in Spain also yielded negative results [24].

Regarding the composition of the patient groups, investigated by Gervasini et al., they were characterised by various histopathologic types of lung cancer, with a significant predominance of squamous cell carcinoma and a low rate of adenocarcinoma; this is inconsistent with the general histopathologic pattern of lung cancer types [25]. This may indicate the presence of some additional factor/factors with carcinogenic potential in this population. In Gemignani et al., the presence of heterozygous CT at that locus was associated with an increased risk of lung cancer, with equal numbers of adenocarcinoma and squamous cell carcinoma [19]. Finally, Sinués et al. report no correlation between SNP C3435T and lung cancer incidence, neither for the total group of lung cancer patients nor after stratification according to histological type [24].

It should be noted that, in line with the findings from all studies given above, no correlation between the presence of C3435T and NSCLC histological type was found in the present study. Moreover, meta-analysis data indicates that the C3435T polymorphism does not contribute to lung cancer risk among an Asian population [23]. Similar conclusions were drawn for studies based on gastrointestinal or ovarian cancer patients, regardless of ethnic group [16, 21, 23, 26]. Although further analysis is needed to verify whether C3435T affects the risk of lung cancer development in Caucasian populations, it seems that both cancer tissue and the patient population influence the role of this synonymous polymorphism in the pathogenesis of cancer.

The C3435T polymorphism demonstrated strong linkage disequilibrium with the other studied forms, one of which is the nonsynonymous G2677T/A, which has been investigated in comparatively small numbers in cancer research. Our findings suggest that G2677T/A has no effect on NSCLC risk, which is in line with the hypothesis that G2677T/A only enhances the effect observed for synonymous SNPs and has little impact on biological phenotype when analyzed separately. No difference in the frequency of G2677T/a genotypes was observed between patients and controls in a study of lung cancer in Eastern European countries [19]. In contrast, Gervasini et al. suggest that the 2677T variant can be crucial in conferring susceptibility to lung cancer in the area of southwest Spain; however, this group demonstrated a lower presence of G allele than in the population of Polish patients with lung cancer [25].

The third most frequently observed polymorphism in the *ABCB1* gene is the nonsynonymous C1236T. In what appears to be the first study to examine this SNP in the context of early onset lung cancer, Gemignani et al. report it to not be associated with the susceptibility of lung cancer [19]. Our present data indicate that heterozygous (CT) occurs significantly more frequently in healthy subjects than in cancer patients. Unfortunately, due to the limited literature base, it is not possible to gather a sufficient amount of data to verify our findings. The present study is also the first to evaluate the distribution of genotypes and allelic variants of the SNP T-129C in the gene promoter region among NSCLC individuals. Although T-129C was not found to influence lung cancer pathogenesis, the low frequencies of the C allele in the general population may contribute to negative outcomes.

After correlating the *ABCB1* genotyping results with age, gender, smoking, histological type of cancer, tumour stage, and grade status, none of the genetic polymorphisms were found to have any influence on lung cancer phenotype. In contrast, Subhani et al. indicated that the homozygous TT of SNP C3435T showed significant association with advanced lung tumour stage, and interestingly, that lung cancer subjects who smoke cigarettes demonstrated more frequent occurrence of 3435TT compared to nonsmokers [22]. Despite this, other studies report no statistically significant correlation between this SNP and smoking status [19, 24, 25]. For the C1236T, a marginally significant correlation was observed between 1236 CT genotype and the age at recruitment to the surgical resection of lung cancer; however, this observation has not been confirmed elsewhere.

In the study presented here, a higher frequency of -T_−129_-C_1236_-T_2677_-T_3435_ haplotype was noted in NSCLC patients than in healthy controls. Unfortunately, again, due to the paucity of studies currently assessing the *ABCB1* haplotype in NSCLC, it is not possible to draw reliable conclusions [25]. However, studies on small cell lung cancer patients have noted that T/A_2677_-T_3435_ carriers showed significantly higher survival probability after chemotherapy [27], which is consistent with our present findings. The prognostic role of *ABCB1* polymorphisms and haplotype is also supported by studies on, *inter alia,* colorectal [28] and breast cancer [29]. Thus, the presence of mutated variants of particular SNPs may be associated with the rick of a malignant neoplasm of epithelial origin.

The linkage between the C3435T SNP and other mutations in the coding region of the *ABCB1* gene or its promoter/enhancer may account for the direct contribution of the silent mutation to disease predisposition. The T variant of SNP C3435T has been associated with lower mRNA stability, diminished P-gp function, and decreased removal of xenobiotics. For G2677T/A, the presence of the T or A variant was connected with transport-related structural changes of P-gp. Finally, C1236T is reported to affect *ABCB1* mRNA stability [17], while the presence of −129TT genotype predisposes to greater DNA damage in subjects exposed to toxic compounds [30]. When combined, such small changes may result in an important reduction of the cellular protective mechanism.

In addition to pumping xenobiotics outside the cell, P-gp inhibits tumourigenesis by working synergistically with immune function and being widely expressed in immune system cells. There is evidence that this protein plays a role in the regulation of cell activity, secretion of cytokines, cytotoxic function, and survival by T, natural killer (NK), and B lymphocytes [31, 32]. Moreover, cancer patients demonstrate changes in the composition and characteristics of blood immune cell subpopulations as a result of interaction between immune system cells and the tumour microenvironment [32]. Additionally, increased activity of these immune cell subpopulations is commonly associated with improved prognosis [33]. However, neutrophil and platelet activation have been found to correlate with reduced patient survival [33, 34]. Therefore, the NLR, LMR, and PLR in cancer patients may be a useful prognostic marker. Therefore, the present study examines whether genotypes/allelic variants of *ABCB1* polymorphisms directly influence blood morphology parameters or the indicators based on them. Our present findings indicate that this is probably not the case. Further analyses are essential to better understand the characteristic pattern presented in the immune system of lung cancer patients. PLR and NLR have been shown to be associated with greater survival, including among patients with NSCLC [35].

The results of the present study should be interpreted with caution because this research is not without limitations. The first is the small sample size. Furthermore, in the present study, the majority of patients enrolled to the study were lost to follow-up and the data about survival time after surgery was incomplete. Therefore, it is impossible to assess the influence of *ABCB1* polymorphisms on survival, this being another limitation of this study.

In conclusion, our present findings indicate that the presence of SNP C3435T of the *ABCB1* gene, either combined with G2677T/A, C1236T, or T-129C or as an independent factor, appears to be associated with NSCLC risk. The remaining three loci in this haplotype appear to be marginally significantly associated with the presence of NSCLC in this study population. Our present study also investigated the potential association of ABCB1 -129/1236/2677/3435 haplotype with NSCLC development. Our findings indicate that the lung cancer and control groups demonstrate significantly different patterns of haplotype distribution. Of particular note is that a higher frequency of -T-129-C1236-T2677-T3435 haplotype was noted in NSCLC patients than in healthy controls. The investigated polymorphisms were not found to be related to the demographic characteristics of lung cancer patients, tumour histopathological results, or blood morphology indices. However, further studies are needed to better understand the effect of genetic variability and mechanisms on lung cancer development. Nevertheless, the presented results provide further evidence that *ABCB1* gene polymorphisms and their haplotype might be genetic risk factors and potential biomarkers for lung cancer.

## Figures and Tables

**Table 1:** The demographic and clinicopathological characteristics of lung cancer patients and healthy controls.

	Item	NSCLC patients		Healthy controls		*p* value
		*n*	*%*	*n*	*%*	
Sex	Male	56	70.0	39	40.6	0.0001
	Female	24	30.0	57	59.4	

Smoking status	Smoker	43	53.8	*—*	*—*	*—*
	Nonsmoker	37	46.2	*—*	*—*	*—*

Histological type	Squamous cell carcinoma	43	53.8	*—*	*—*	*—*
	Adenocarcinoma	35	43.8	*—*	*—*	*—*
	Adenosquamous carcinoma	2	2.4	*—*	*—*	*—*

TNM stage	I	43	53.8	*—*	*—*	*—*
	II	24	30.0	*—*	*—*	*—*
	III	13	16.2	—	*—*	*—*

Grade	G1	3	3.8	—	—	—
	G2	56	70.9	—	—	—
	G3	20	25.3	—	—	—

**Table 2:** Genotype distribution and allele frequencies of *ABCB1* SNPs in non-small cell lung carcinoma (NSCLC) patients and control subjects.

*ABCB1*								
SNPs ID		Genotype				Allele		
		WT	HET	MUT	*p* value	WT	MUT	*p* value
T-129C	Study	76 (95.0%)	4 (5.0%)	0 (0.0%)	0.8970	156 (97.5%)	4 (2.5%)	0.8175
	Control	90 (95.7%)	4 (4.3%)	0 (0.0%)		184 (97.9%)	4 (2.1%)	
	OR (95%Cl)	HET: 1.18 (0.29–4.90)			0.8154	MUT: 1.18 (0.29–4.79)		
		MUT: NA			NA			

C1236T	Study	**30 (37.5%)**	**29 (36.3%)**	**21 (26.2%)**	**0.0223**	89 (55.6%)	71 (44.4%)	0.7535
	Control^*∗*^	**28 (29.2%)**	**54 (56.3%)**	**14 (14.5%)**		110 (57.3%)	82 (42.7%)	
	OR (95% CI)	**HET: 0.50 (0.25-0.99)**			**0.0480**	MUT: 1.07 (0.70–1.63)		
		MUT: 1.40 (0.60–3.28)			0.4379			

G2677T/A	Study	25 (32.1%)	33 (42.3%)	20 (25.6%)	0.1116	83 (53.2%)	73 (46.8%)	0.2104
	Control^*∗*^	32 (33.4%)	51 (53.1%)	13 (13.5%)		115 (59.9%)	77 (40.1%)	
	OR (95% CI)	HET: 0.83 (0.42–1.64)			0.5882	1.31 (0.86–2.01)		
		MUT: 1.97 (0.82–4.71)			0.1280			

C3435T	Study	**11 (13.7%)**	**41 (51.3%)**	**28 (35.0%)**	**0.0319**	**63 (39.4%)**	**97 (60.6%)**	**0.0103**
	Control^*∗*^	**27 (28.1%)**	**48 (50.0%)**	**21 (21.9%)**		**102 (53.1%)**	**90 (46.9%)**	
	OR (95% CI)	HET: 2.10 (0.93–4.74)			0.0752	**1.75 (1.14–2.67)**		
		**MUT: 3.27 (1.33-8.06)**			**0.0099**			

^*∗*^Frequencies taken from publication [8]. *P* < 0.05 indicates statistical significance and bold indicate a significant difference.

## Data Availability

The data used to support the findings of this study are available from the corresponding author on reasonable request.

## Abbreviations

*ABCB1*: ATP binding cassette subfamily B member 1
LD: Linkage disequilibrium
LMR: Lymphocyte-to-monocyte ratio
NLR: Neutrophil-to-lymphocyte ratio
NSCLC: Non-small cell lung cancer
OR: Odds ratio
P-gp: P-glycoprotein
PLR: Platelet-to-lymphocyte ratio
RFLP method: Restriction fragment length polymorphism method
SeqPCR: Sequencing polymerase chain reaction
SNP: Single nucleotide polymorphism
95% CI: 95% confidence interval.

## Supplementary Materials

Supplementary Materials Supplement Figure 1: examples of a separation of *ABCB1* fragment. Sanger sequencing chromatograms showing polymorphism C1236T in (a) CT heterozygous, (b) CC homozygous, and (c) TT homozygous. Supplement Figure 2: examples of a separation of *ABCB1* fragment. Sanger sequencing chromatograms showing polymorphism G2677T/A in (a) TT homozygous, (b) GG homozygous, (c) GT heterozygous, (d) TA heterozygous, and (e) GA heterozygous. Supplement Table A1: genotype frequencies of *ABCB1* gene T-129C polymorphism according to clinicopathological parameters. Supplement Table A2: genotype and allele frequencies of *ABCB1* gene C1236T polymorphism according to clinicopathological parameters. Supplement Table A3: genotype and allele frequencies of *ABCB1* gene G2677T/A polymorphism according to clinicopathological parameters. Supplement Table A4: genotype and allele frequencies of *ABCB1* gene C3435T polymorphism according to clinicopathological parameters. Supplement Table B1: characterization of age at lung cancer disease onset and blood morphology indices according to T-129C *ABCB1* genotype. Supplement Table B2: characterization of age at lung cancer disease onset and blood morphology indices according to C1236T *ABCB1* genotype and allele status. Supplement Table B3: characterization of age at lung cancer disease onset and blood morphology indices according to G2677T/A *ABCB1* genotype and allele status. Supplement Table B4: characterization of age at lung cancer disease onset and blood morphology indices according to C3435T *ABCB1* genotype and allele status.
