# Supplementary material for: The Interaction between Four Polymorphisms and Haplotype of ABCB1, the Risk of Non-Small Cell Lung Cancer, and the Disease Phenotype
Source: J Oncol. 2023 Jan 24;2023:7925378. doi: 10.1155/2023/7925378 (PMC9902128; doi:10.1155/2023/7925378)
Supplement: Supplementary Materials — Supplement Figure 1: examples of a separation of ABCB1 fragment. Sanger sequencing chromatograms showing polymorphism C1236T in (a) CT heterozygous, (b) CC homozygous, and (c) TT homozygous. Supplement Figure 2: examples of a separation of ABCB1 fragment. Sanger sequencing chromatograms showing polymorphism G2677T/A in (a) TT homozygous, (b) GG homozygous, (c) GT heterozygous, (d) TA heterozygous, and (e) GA heterozygous. Supplement Table A1: genotype frequencies of ABCB1 gene T-129C polymorphism according to clinicopathological parameters. Supplement Table A2: genotype and allele frequencies of ABCB1 gene C1236T polymorphism according to clinicopathological parameters. Supplement Table A3: genotype and allele frequencies of ABCB1 gene G2677T/A polymorphism according to clinicopathological parameters. Supplement Table A4: genotype and allele frequencies of ABCB1 gene C3435T polymorphism according to clinicopathological parameters. Supplement Table B1: characterization of age at lung cancer disease onset and blood morphology indices according to T-129C ABCB1 genotype. Supplement Table B2: characterization of age at lung cancer disease onset and blood morphology indices according to C1236T ABCB1 genotype and allele status. Supplement Table B3: characterization of age at lung cancer disease onset and blood morphology indices according to G2677T/A ABCB1 genotype and allele status. Supplement Table B4: characterization of age at lung cancer disease onset and blood morphology indices according to C3435T ABCB1 genotype and allele status. [file 7925378.f1.zip › Supplementary_materials_A.docx]

Supplement table A1. Genotype frequencies of *ABCB1* gene T-129C polymorphism according to clinicopathological parameters.

| **Feature** | | **T-129C** | | **P value** |
| --- | --- | --- | --- | --- |
|  |  | **TT, n (%)** | **CT, n (%)** |  |
| **gender** | **women** | 24 (100.0) | 0 (0.0) | 0.4333* |
|  | **men** | 52 (92.9) | 4 (7.1) |  |
| **smoking** | **yes** | 43 (100.0) | 0 (0.0) | 0.0896* |
|  | **no** | 33 (89.2) | 4 (10.8) |  |
| **histological type** | **adenomatous or adenosqamous** | 34 (91.9) | 3 (8.1) | 0.5036* |
|  | **squamous** | 42 (97.7) | 1 (2.3) |  |
| **TNM stage** | **I** | 41 (95.3) | 2 (4.7) | 0.7188* |
|  | **II or III** | 35 (94.6) | 2 (5.4) |  |
| **grade** | **G1 or G2** | 59 (98.3) | 1 (1.7) | 0.0756* |
|  | **G3** | 17 (85.0) | 3 (15.0) |  |

*Pearson’s χ^2^ with Yates’ correction

Supplement table A2. Genotype and allele frequencies of *ABCB1* gene C1236T polymorphism according to clinicopathological parameters.

| **Feature** | | **1236** | | | **P value** | **C 1236** | | **P value** | **T 1236** | | **P value** |
| --- | --- | --- | --- | --- | --- | --- | --- | --- | --- | --- | --- |
|  |  | **CC** | **CT** | **TT** |  | **present** | **absent** |  | **present** | **absent** |  |
| **gender** | **women** | 9 (37.5) | 8 (33.3) | 7 (29.2) | 0.9087* | 17 (70.8) | 7 (29.2) | 0.6997** | 15 (62.5) | 9 (37.5) | 1.0000** |
|  | **men** | 21 (37.5) | 21 (37.5) | 14 (25.0) |  | 42 (75.0) | 14 (25.0) |  | 35 (62.5) | 21 (37.5) |  |
| **smoking** | **yes** | 14 (32.6) | 15 (34.9) | 14 (32.6) | 0.3565* | 29 (67.4) | 14 (32.6) | 0.1695** | 29 (67.4) | 14 (32.6) | 0.3250* |
|  | **no** | 16 (43.2) | 14 (37.8) | 7 (18.9) |  | 30 (81.1) | 7 (18.9) |  | 21 (56.8) | 16 (43.2) |  |
| **histological type** | **adenomatous or adenosqamous** | 13 (35.1) | 13 (35.1) | 11 (29.7) | 0.8010* | 26 (70.3) | 11 (29.7) | 0.5144** | 24 (64.9) | 13 (35.1) | 0.6853* |
|  | **squamous** | 17 (39.5) | 16 (37.2) | 10 (23.3) |  | 33 (76.7) | 10 (23.3) |  | 26 (60.5) | 17 (39.5) |  |
| **TNM stage** | **I** | 15 (34.9) | 18 (41.9) | 10 (23.3) | 0.5235* | 33 (76.7) | 10 (23.3) | 0.5144** | 28 (65.1) | 15 (34.9) | 0.6023* |
|  | **II or III** | 15 (40.5) | 11 (29.7) | 11 (29.7) |  | 26 (70.3) | 11 (29.7) |  | 15 (40.5) | 22 (59.5) |  |
| **grade** | **G1 or G2** | 24 (40.0) | 18 (30.0) | 18 (30.0) | 0.1181* | 42 (70.0) | 18 (30.0) | 0.1895** | 36 (60.0) | 24 (40.0) | 0.4266** |
|  | **G3** | 6 (30.0) | 11 (55.0) | 3 (15.0) |  | 17 (85.0) | 3 (15.0) |  | 14 (70.0) | 6 (30.0) |  |

* Pearson’s χ^2^ test; ** V^2^ test;

Supplement table A3. Genotype and allele frequencies of *ABCB1* gene G2677T/A polymorphism according to clinicopathological parameters.

| **Feature** | | **2677** | | | **P value** | **G 2677** | | **P value** | **T or A 2677** | | **P value** |
| --- | --- | --- | --- | --- | --- | --- | --- | --- | --- | --- | --- |
|  |  | **GG** | **GT or GA** | **TT or TA** |  | **present** | **absent** |  | **present** | **absent** |  |
|  |  |  |  |  |  |  |  |  |  |  |  |
| **gender** | **women** | 7 (30.4) | 7 (30.4) | 9 (39.1) | 0.1801* | 14 (60.9) | 9 (39.1) | 0.0796** | 16 (69.6) | 7 (30.4) | 0.8442** |
|  | **men** | 18 (32.7) | 26 (47.3) | 11 (20.0) |  | 44 (80.0) | 11 (20.0) |  | 37 (67.3) | 18 (32.7) |  |
| **smoking** | **yes** | 14 (34.1) | 16 (39.0) | 11 (26.8) | 0.8244* | 30 (73.2) | 11 (26.8) | 0.8015** | 27 (65.9) | 14 (34.1) | 0.6764* |
|  | **no** | 11 (29.7) | 17 (45.9) | 9 (24.3) |  | 28 (75.7) | 9 (24.3) |  | 26 (70.3) | 11 (29.7) |  |
| **histological type** | **adenomatous or adenosquamous** | 11 (30.6) | 12 (33.3) | 13 (36.1) | 0.1238* | 23 (63.9) | 13 (36.1) | 0.0514** | 25 (69.4) | 11 (30.6) | 0.7933* |
|  | **squamous** | 14 (33.3) | 21 (50.0) | 7 (16.7) |  | 35 (83.3) | 7 (16.7) |  | 28 (67.7) | 14 (33.3) |  |
| **TNM stage** | **I** | 13 (37.7) | 19 (46.3) | 9 (22.0) | 0.6722* | 32 (78.0) | 9 (22.0) | 0.4251** | 28 (68,3) | 13 (31.7) | 0.9454* |
|  | **II or III** | 12 (32.4) | 14 (37.8) | 11 (29.7) |  | 26 (70.3) | 11 (29.7) |  | 25 (67.6) | 12 (32.4) |  |
| **grade** | **G1 or G2** | 21 (35.6) | 21 (35.6) | 17 (28.8) | 0.1065* | 42 (71.2) | 17 (28.8) | 0.4073# | 38 (64.4) | 21 (35.6) | 0.2406** |
|  | **G3** | 4 (21.1) | 12 (63.2) | 3 (15.8) |  | 16 (84.2) | 3 (15.8) |  | 15 (78.9 ) | 4 (21.1) |  |

* Pearson’s χ^2^ test; ** V^2^ test; # Pearson’s χ^2^ test with Yates’ correction

Supplement table A4. Genotype and allele frequencies of *ABCB1* gene C3435T polymorphism according to clinicopathological parameters.

| **Feature** | | **3435** | | | **P value** | **C 3435** | | **P value** | **T 3435** | | **P value** |
| --- | --- | --- | --- | --- | --- | --- | --- | --- | --- | --- | --- |
|  |  | **CC** | **CT** | **TT** |  | **present** | **absent** |  | **present** | **absent** |  |
| **gender** | **women** | 5 (20.8) | 12 (50.0) | 7 (29.2) | 0.4505* | 17 (70.8) | 7 (29.2) | 0.4767** | 19 (79.2) | 5 (20.8) | 0.3952*** |
|  | **men** | 6 (10.7) | 29 (51.8) | 21 (37.5) |  | 35 (62.5) | 21 (37.5) |  | 50 (89.3) | 6 (10.7) |  |
| **smoking** | **yes** | 4 (9.3) | 19 (44.2) | 20 (46.5) | **0.0561*** | 23 (53.5) | 20 (46.5) | **0.0200*** | 39 (90.7) | 4 (9.3) | 0.2195** |
|  | **no** | 7 (18.9) | 22 (59.5) | 8 (21.6) |  | 29 (78.4) | 8 (21.6) |  | 30 (81.1) | 7 (18.9) |  |
| **histological type** | **adenomatous or adenosquamous** | 6 (16.2) | 20 (54.1) | 11 (29.7) | 0.6199* | 26 (70.3) | 11 (29.7) | 0.3593* | 31 (83.8) | 6 (16.2) | 0.5549** |
|  | **squamous** | 5 (11.6) | 21 (48.8) | 17 (39.5) |  | 26 (60.5) | 17 (39.5) |  | 38 (88.4) | 5 (11.6) |  |
| **TNM stage** | **I** | 7 (16.3) | 21 (48.8) | 15 (34.9) | 0.7640* | 28 (65.1) | 15 (34.9) | 0.9813* | 36 (83.7) | 7 (16.3) | 0.4816** |
|  | **II or III** | 4 (10.8) | 20 (54.1) | 13 (35.1) |  | 24 (64.9) | 13 (35.1) |  | 33 (89.2) | 4 (10.8) |  |
| **grade** | **G1 or G2** | 9 (15.0) | 28 (46.7) | 23 (38.3) | 0.3645* | 37 (61.7) | 23 (38.3) | 0.2820 | 51 (85.0) | 9 (15.0) | 0.8513*** |
|  | **G3** | 2 (10.0) | 13 (65.0) | 5 (25.0) |  | 15 (75.0) | 5 (25.0) |  | 18 (90.0) | 2 (10.0) |  |

*Pearson χ^2^ test; ** V^2^ test; χ2 test with Yates’ correction
