# Supplementary material for: The Interaction between Four Polymorphisms and Haplotype of ABCB1, the Risk of Non-Small Cell Lung Cancer, and the Disease Phenotype
Source: J Oncol. 2023 Jan 24;2023:7925378. doi: 10.1155/2023/7925378 (PMC9902128; doi:10.1155/2023/7925378)
Supplement: Supplementary Materials — Supplement Figure 1: examples of a separation of ABCB1 fragment. Sanger sequencing chromatograms showing polymorphism C1236T in (a) CT heterozygous, (b) CC homozygous, and (c) TT homozygous. Supplement Figure 2: examples of a separation of ABCB1 fragment. Sanger sequencing chromatograms showing polymorphism G2677T/A in (a) TT homozygous, (b) GG homozygous, (c) GT heterozygous, (d) TA heterozygous, and (e) GA heterozygous. Supplement Table A1: genotype frequencies of ABCB1 gene T-129C polymorphism according to clinicopathological parameters. Supplement Table A2: genotype and allele frequencies of ABCB1 gene C1236T polymorphism according to clinicopathological parameters. Supplement Table A3: genotype and allele frequencies of ABCB1 gene G2677T/A polymorphism according to clinicopathological parameters. Supplement Table A4: genotype and allele frequencies of ABCB1 gene C3435T polymorphism according to clinicopathological parameters. Supplement Table B1: characterization of age at lung cancer disease onset and blood morphology indices according to T-129C ABCB1 genotype. Supplement Table B2: characterization of age at lung cancer disease onset and blood morphology indices according to C1236T ABCB1 genotype and allele status. Supplement Table B3: characterization of age at lung cancer disease onset and blood morphology indices according to G2677T/A ABCB1 genotype and allele status. Supplement Table B4: characterization of age at lung cancer disease onset and blood morphology indices according to C3435T ABCB1 genotype and allele status. [file 7925378.f1.zip › Supplementary_materials_B.docx]

**Supplement table B1.** Characterization of age at the lung cancer disease onset and blood morphology indices according to T-129C *ABCB1* genotype

|  | **T-129C** | **N** | **Mean** | **Median** | **Min.** | **Max.** | **IQR** | **SD** | **CV** | **P value** |
| --- | --- | --- | --- | --- | --- | --- | --- | --- | --- | --- |
| **Age** | **TT** | 76 | 67.171 | 68.000 | 32.000 | 82.000 | 9.000 | 8.169 | 12.161 | 0.5810* |
|  | **CT** | 4 | 70.250 | 68.000 | 66.000 | 79.000 | 6.500 | 5.909 | 8.411 |  |
| **WBC** | **TT** | 76 | 9.545 | 9.070 | 5.210 | 19.390 | 3.415 | 2.914 | 30.533 | 0.9560* |
|  | **CC** | 4 | 9.853 | 10.085 | 4.540 | 14.700 | 8.115 | 4.839 | 49.116 |  |
| **RBC** | **TT** | 76 | 4.438 | 4.535 | 2.400 | 5.310 | 0.795 | 0.537 | 12.09 | 0.0956* |
|  | **CT** | 4 | 4.018 | 4.050 | 3.540 | 4.430 | 0.775 | 0.454 | 11.30 |  |
| **HTC** | **TT** | 76 | 39.301 | 39.500 | 22.300 | 47.700 | 5.900 | 4.408 | 11.216 | 0.3099* |
|  | **CT** | 4 | 37.425 | 36.050 | 33.300 | 44.300 | 6.750 | 4.871 | 13.016 |  |
| **Ig** | **TT** | 71 | 0.197 | 0.100 | 0 | 1.050 | 0.250 | 0.215 | 108.795 | 0.0748* |
|  | **CT** | 3 | 0.037 | 0.050 | 0.010 | 0.050 | 0.040 | 0.023 | 62.984 |  |
| **Neutro** | **TT** | 76 | 6.711 | 6.315 | 2.220 | 16.570 | 3.330 | 2.756 | 41.071 | 0.7996* |
|  | **CT** | 4 | 7.118 | 7.480 | 2.550 | 10.960 | 6.595 | 3.971 | 55.796 |  |
| **Limfo** | **TT** | 76 | 1.853 | 1.840 | 0.010 | 4.870 | 0.940 | 0.844 | 45.542 | 0.3893* |
|  | **CT** | 4 | 1.758 | 1.170 | 1.050 | 3.640 | 1.315 | 1.256 | 71.487 |  |
| **Mono** | **TT** | 76 | 0.754 | 0.670 | 0 | 1.550 | 0.420 | 0.307 | 40.709 | 0.2510* |
|  | **CT** | 4 | 0.527 | 0.555 | 0.078 | 0.920 | 0.486 | 0.349 | 66.284 |  |
| **PLT** | **TT** | 76 | 268.434 | 261.000 | 109.000 | 617.000 | 94.000 | 88.070 | 32.809 | 0.5292* |
|  | **CT** | 4 | 309.750 | 299.500 | 104.000 | 536.000 | 243.500 | 178.180 | 57.5239 |  |
| **NLR** | **TT** | 75 | 4.659 | 3.520 | 0.787 | 29.347 | 2.931 | 4.271 | 91.670 | 0.8668* |
|  | **CT** | 4 | 4.694 | 3.569 | 2.429 | 9.210 | 4.248 | 3.137 | 66.837 |  |
| **LMR** | **TT** | 74 | 2.576 | 2.580 | 0.020 | 5.610 | 1.767 | 1.235 | 47.956 | 0.4334** |
|  | **CT** | 3 | 3.170 | 2.350 | 1.290 | 5.870 | 4.580 | 2.398 | 75.634 |  |
| **PLR** | **TT** | 75 | 182.742 | 149.677 | 47.000 | 976.000 | 95.000 | 125.174 | 68.497 | 0.4404* |
|  | **CT** | 4 | 268.762 | 210.500 | 99.048 | 555.000 | 291.476 | 204.604 | 76.128 |  |

*Mann-Whitney U test; ** Student’s t test

**Supplement table B2.** Characterization of age at the lung cancer disease onset and blood morphology indices according to C1236T *ABCB1* genotype and allele status

|  | **1236** | **N** | **Mean** | **Median** | **Min.** | **Max.** | **IQR** | **SD** | **CV** | **P value** |
| --- | --- | --- | --- | --- | --- | --- | --- | --- | --- | --- |
| **Age** | **CC** | 30 | 66,133 | 67,000 | 53,000 | 76,000 | 5,000 | 5,888 | 8,904 | **0.0410**** |
|  | **CT** | 29 | 70,586 | 70,000 | 57,000 | 82,000 | 10,000 | 7,1340 | 10,107 |  |
|  | **TT** | 21 | 64,524 | 66,000 | 32,000 | 80,000 | 10,000 | 10,486 | 16,252 |  |
|  | **C present** | 59 | 68,322 | 68,000 | 53,000 | 82,000 | 9,000 | 6,852 | 10,029 | 0.2248# |
|  | **C absent** | 21 | 64,524 | 66,000 | 32,000 | 80,000 | 10,000 | 10,486 | 16,252 |  |
|  | **T present** | 50 | 68,040 | 70,000 | 32,000 | 82,000 | 10,000 | 9,116 | 13,398 | 0.1641# |
|  | **T absent** | 30 | 66,133 | 67,000 | 53,000 | 76,000 | 5,000 | 5,888 | 8,904 |  |
| **WBC** | **CC** | 30 | 9,382 | 8,695 | 4,540 | 19,390 | 4,0900 | 3,564 | 37,993 | 0.2305** |
|  | **CT** | 29 | 9,133 | 8,820 | 5,210 | 13,120 | 3,620 | 2,319 | 25,395 |  |
|  | **TT** | 21 | 10,405 | 10,200 | 5,760 | 18,080 | 2,380 | 2,892 | 27,798 |  |
|  | **C present** | 59 | 9,260 | 8,820 | 4,540 | 19,390 | 3,920 | 2,994 | 32,336 | 0.0911# |
|  | **C absent** | 21 | 10,405 | 10,200 | 5,760 | 18,080 | 2,380 | 2,892 | 27,798 |  |
|  | **T present** | 50 | 9,668 | 9,450 | 5,210 | 18,080 | 3,500 | 2,625 | 27,154 | 0.3179# |
|  | **T absent** | 30 | 9,382 | 8,695 | 4,540 | 19,390 | 4,090 | 3,564 | 37,993 |  |
| **RBC** | **CC** | 30 | 4,420 | 4,425 | 3,460 | 5,310 | 0,680 | 0,497 | 11,248 | 0.9660** |
|  | **CT** | 29 | 4,398 | 4,430 | 2,400 | 5,290 | 0,660 | 0,578 | 13,145 |  |
|  | **TT** | 21 | 4,440 | 4,590 | 2,970 | 5,180 | 0,990 | 0,563 | 12,682 |  |
|  | **C present** | 59 | 4,409 | 4,430 | 2,400 | 5,310 | 0,710 | 0,534 | 12,109 | 0.8015# |
|  | **C absent** | 21 | 4,440 | 4,590 | 2,970 | 5,180 | 0,990 | 0,563 | 12,682 |  |
|  | **T present** | 50 | 4,416 | 4,485 | 2,400 | 5,290 | 0,810 | 0,566 | 12,828 | 0.8697# |
|  | **T absent** | 30 | 4,420 | 4,425 | 3,460 | 5,310 | 0,680 | 0,497 | 11,248 |  |
| **HTC** | **CC** | 30 | 39,030 | 39,550 | 32,400 | 45,600 | 4,800 | 3,712 | 9,512 | 0.8989** |
|  | **CT** | 29 | 39,207 | 39,500 | 22,300 | 45,900 | 5,400 | 4,790 | 12,218 |  |
|  | **TT** | 21 | 39,462 | 38,000 | 28,500 | 47,700 | 6,900 | 4,988 | 12,639 |  |
|  | **C present** | 59 | 39,117 | 39,500 | 22,300 | 45,900 | 4,900 | 4,240 | 10,839 | 0.9477# |
|  | **C absent** | 21 | 39,462 | 38,000 | 28,500 | 47,700 | 6,900 | 4,988 | 12,639 |  |
|  | **T present** | 50 | 39,314 | 38,950 | 22,300 | 47,700 | 6,900 | 4,825 | 12,273 | 0.6655# |
|  | **T absent** | 30 | 39,030 | 39,550 | 32,400 | 45,600 | 4,800 | 3,712 | 9,512 |  |
| **Ig** | **CC** | 28 | 0,209 | 0,1150 | 0,002 | 0,700 | 0,240 | 0,200 | 95,608 | 0,3232** |
|  | **CT** | 27 | 0,212 | 0,100 | 0,000 | 1,050 | 0,260 | 0,256 | 120,682 |  |
|  | **TT** | 19 | 0,133 | 0,060 | 0,000 | 0,600 | 0,160 | 0,155 | 116,074 |  |
|  | **C present** | 55 | 0,211 | 0,110 | 0 | 1,050 | 0,250 | 0,227 | 107,796 | 0.1855# |
|  | **C absent** | 19 | 0,133 | 0,060 | 0 | 0,600 | 0,160 | 0,155 | 116,074 |  |
|  | **T present** | 46 | 0,180 | 0,075 | 0 | 1,050 | 0,210 | 0,221 | 123,281 | 0.2223# |
|  | **T absent** | 28 | 0,209 | 0,115 | 0,002 | 0,700 | 0,240 | 0,200 | 95,608 |  |
| **Neutro** | **CC** | 30 | 6,702 | 6,035 | 2,220 | 16,570 | 3,810 | 3,317 | 49,496 | 0,3012** |
|  | **CT** | 29 | 6,330 | 5,460 | 3,300 | 11,420 | 3,490 | 2,308 | 36,470 |  |
|  | **TT** | 21 | 7,328 | 6,970 | 3,660 | 15,600 | 3,210 | 2,619 | 35,746 |  |
|  | **C present** | 59 | 6,519 | 6,020 | 2,220 | 16,570 | 3,980 | 2,848 | 43,684 | 0.1272# |
|  | **C absent** | 21 | 7,328 | 6,970 | 3,660 | 15,600 | 3,210 | 2,619 | 35,746 |  |
|  | **T present** | 50 | 6,749 | 6,615 | 3,300 | 15,600 | 3,210 | 2,468 | 36,575 | 0.6228# |
|  | **T absent** | 30 | 6,702 | 6,035 | 2,220 | 16,570 | 3,810 | 3,317 | 49,496 |  |
| **Limfo** | **CC** | 30 | 1,770 | 1,565 | 0,490 | 3,640 | 0,960 | 0,792 | 44,740 | 0,7765** |
|  | **CT** | 29 | 1,855 | 1,930 | 0,560 | 4,870 | 0,850 | 0,830 | 44,743 |  |
|  | **TT** | 21 | 1,950 | 1,860 | 0,010 | 4,650 | 1,040 | 1,007 | 51,658 |  |
|  | **C present** | 59 | 1,812 | 1,800 | 0,490 | 4,870 | 0,930 | 0,805 | 44,429 | 0.5153# |
|  | **C absent** | 21 | 1,950 | 1,860 | 0,010 | 4,650 | 1,040 | 1,007 | 51,658 |  |
|  | **T present** | 50 | 1,895 | 1,920 | 0,010 | 4,870 | 0,980 | 0,900 | 47,497 | 0.5881# |
|  | **T absent** | 30 | 1,770 | 1,565 | 0,490 | 3,640 | 0,960 | 0,792 | 44,740 |  |
| **Mono** | **CC** | 30 | 0,699 | 0,620 | 0 | 1,430 | 0,330 | 0,333 | 47,647 | 0,4448** |
|  | **CT** | 29 | 0,753 | 0,710 | 0,390 | 1,550 | 0,390 | 0,272 | 36,109 |  |
|  | **TT** | 21 | 0,791 | 0,730 | 0,010 | 1,460 | 0,420 | 0,334 | 42,258 |  |
|  | **C present** | 59 | 0,726 | 0,620 | 0,000 | 1,550 | 0,420 | 0,303 | 41,781 | 0.2814# |
|  | **C absent** | 21 | 0,791 | 0,730 | 0,010 | 1,460 | 0,420 | 0,334 | 42,258 |  |
|  | **T present** | 50 | 0,769 | 0,715 | 0,010 | 1,550 | 0,410 | 0,297 | 38,619 | 0.2765# |
|  | **T absent** | 30 | 0,699 | 0,620 | 0,000 | 1,430 | 0,330 | 0,333 | 47,647 |  |
| **PLT** | **CC** | 30 | 285,867 | 264,000 | 104,000 | 617,000 | 108,000 | 111,507 | 39,007 | 0,5037** |
|  | **CT** | 29 | 249,793 | 250,000 | 122,000 | 372,000 | 83,000 | 62,463 | 25,006 |  |
|  | **TT** | 21 | 277,143 | 284,000 | 109,000 | 475,000 | 127,000 | 98,280 | 35,462 |  |
|  | **C present** | 59 | 268,136 | 254,000 | 104,000 | 617,000 | 95,000 | 91,822 | 34,245 | 0.5921# |
|  | **C absent** | 21 | 277,143 | 284,000 | 109,000 | 475,000 | 127,000 | 98,280 | 35,462 |  |
|  | **T present** | 50 | 261,280 | 261,000 | 109,000 | 475,000 | 99,000 | 79,736 | 30,518 | 0.5055# |
|  | **T absent** | 30 | 285,867 | 264,000 | 104,000 | 617,000 | 108,000 | 111,507 | 39,007 |  |
| **NLR** | **CC** | 30 | 5,039 | 3,319 | 0,787 | 29,347 | 2,388 | 5,321 | 105,593 | 0,9683** |
|  | **CT** | 29 | 4,399 | 3,833 | 0,979 | 20,393 | 3,419 | 3,698 | 84,072 |  |
|  | **TT** | 20 | 4,473 | 3,769 | 1,494 | 12,893 | 2,857 | 2,977 | 66,546 |  |
|  | **C present** | 59 | 4,724 | 3,476 | 0,787 | 29,347 | 3,042 | 4,567 | 96,681 | 0.8524# |
|  | **C absent** | 20 | 4,473 | 3,769 | 1,494 | 12,893 | 2,857 | 2,977 | 66,546 |  |
|  | **T present** | 49 | 4,429 | 3,832 | 0,979 | 20,393 | 3,006 | 3,389 | 76,520 | 0.9557# |
|  | **T absent** | 30 | 5,039 | 3,319 | 0,787 | 29,347 | 2,388 | 5,321 | 105,593 |  |
| **LMR** | **CC** | 28 | 2,704 | 2,786 | 0,360 | 5,870 | 1,551 | 1,221 | 45,168 | 0,4580** |
|  | **CT** | 29 | 2,693 | 2,530 | 0,780 | 5,098 | 2,093 | 1,310 | 48,650 |  |
|  | **TT** | 20 | 2,317 | 2,340 | 0,020 | 5,610 | 1,485 | 1,327 | 57,261 |  |
|  | **C present**  **C absent**  **T present**  **T absent** | 57 | 2,698 | 2,630 | 0,360 | 5,870 | 1,840 | 1,256 | 46,545 | 0.2532## |
|  |  | 20 | 2,317 | 2,340 | 0,020 | 5,610 | 1,485 | 1,327 | 57,261 |  |
|  |  | 49 | 2,539 | 2,500 | 0,020 | 5,610 | 1,632 | 1,316 | 51,836 | 0.5893## |
|  |  | 28 | 2,704 | 2,786 | 0,360 | 5,870 | 1,551 | 1,221 | 45,168 |  |
| **PLR** | **CC** | 30 | 213,309 | 155,307 | 62,000 | 976,000 | 104,103 | 169,797 | 79,602 | 0,3654* |
|  | **CT** | 29 | 184,348 | 147,500 | 64,000 | 555,000 | 123,000 | 111,939 | 60,722 |  |
|  | **TT** | 20 | 151,769 | 139,500 | 47,000 | 323,864 | 77,550 | 65,053 | 42,863 |  |
|  | **C present** | 59 | 199,074 | 152,000 | 62,000 | 976,000 | 124,000 | 143,798 | 72,234 | 0.2766# |
|  | **C absent** | 20 | 151,769 | 139,500 | 47,000 | 323,864 | 77,550 | 65,053 | 42,863 |  |
|  | **T present** | 49 | 171,050 | 140,000 | 47,000 | 555,000 | 99,000 | 96,158 | 56,216 | 0.1943# |
|  | **T absent** | 30 | 213,309 | 155,307 | 62,000 | 976,000 | 104,103 | 169,797 | 79,602 |  |

* ANOVA; ** Kruskal-Wallis ANOVA; ## Mann-Whitney’s U test; Student’s t test

**Supplement table B3.** Characterization of age at the lung cancer disease onset and blood morphology indices according to G2677T/A *ABCB1* genotype and allele status

|  | **2677** | **N** | **Mean** | **Median** | **Min.** | **Max.** | **IQR** | **SD** | **CV** | **P value*** |
| --- | --- | --- | --- | --- | --- | --- | --- | --- | --- | --- |
| **Age** | **GG** | 25 | 67.960 | 68.000 | 55.000 | 76.000 | 7.000 | 5.481 | 8.065 | 0.7506* |
|  | **GT or GA** | 33 | 68.485 | 68.000 | 53.000 | 82.000 | 8.000 | 7.779 | 11.358 |  |
|  | **TT or TA** | 20 | 64.850 | 67.000 | 32.000 | 80.000 | 11.500 | 10.999 | 16.961 |  |
|  | **G present** | 58 | 68.259 | 68.000 | 53.000 | 82.000 | 8.000 | 6.833 | 10.010 | 0.4535** |
|  | **G absent** | 20 | 64.850 | 67.000 | 32.000 | 80.000 | 11.500 | 10.999 | 16.961 |  |
|  | **T or A present** | 53 | 67.113 | 68.000 | 32.000 | 82.000 | 10.000 | 9.198 | 13.705 | 0.7359** |
|  | **T or A absent** | 25 | 67.960 | 68.000 | 55.000 | 76.000 | 7.000 | 5.481 | 8.065 |  |
| **WBC** | **GG** | 25 | 9.443 | 9.070 | 5.850 | 15.810 | 3.490 | 2.732 | 28.933 | 0.8053* |
|  | **GT or GA** | 33 | 9.520 | 8.980 | 4.540 | 19.390 | 3.530 | 3.104 | 32.600 |  |
|  | **TT or TA** | 20 | 10.080 | 9.390 | 5.500 | 18.080 | 3.560 | 3.209 | 31.832 |  |
|  | **G present** | 58 | 9.487 | 9.070 | 4.540 | 19.390 | 3.620 | 2.924 | 30.825 | 0.5142** |
|  | **G absent** | 20 | 10.080 | 9.390 | 5.500 | 18.080 | 3.560 | 3.209 | 31.832 |  |
|  | **T or A present** | 53 | 9.732 | 9.310 | 4.540 | 19.390 | 3.500 | 3.125 | 32.110 | 0.7889** |
|  | **T or A absent** | 25 | 9.443 | 9.070 | 5.850 | 15.810 | 3.490 | 2.732 | 28.933 |  |
| **RBC** | **GG** | 25 | 4.438 | 4.560 | 3.460 | 5.310 | 0.520 | 0.505 | 11.369 | 0.9108* |
|  | **GT or GA** | 33 | 4.429 | 4.430 | 2.400 | 5.290 | 0.810 | 0.582 | 13.148 |  |
|  | **TT or TA** | 20 | 4.382 | 4.395 | 2.970 | 5.180 | 0.810 | 0.542 | 12.375 |  |
|  | **G present** | 58 | 4.433 | 4.505 | 2.400 | 5.310 | 0.760 | 0.546 | 12.306 | 0.6762** |
|  | **G absent** | 20 | 4.382 | 4.395 | 2.970 | 5.180 | 0.810 | 0.542 | 12.375 |  |
|  | **T or A present** | 53 | 4.411 | 4.430 | 2.400 | 5.290 | 0.810 | 0.563 | 12.757 | 0.9317** |
|  | **T or A absent** | 25 | 4.438 | 4.560 | 3.460 | 5.310 | 0.520 | 0.505 | 11.369 |  |
| **HTC** | **GG** | 25 | 39.332 | 39.800 | 32.400 | 45.600 | 4.900 | 3.693 | 9.390 | 0.8141* |
|  | **GT or GA** | 33 | 39.312 | 39.500 | 22.300 | 46.600 | 6.000 | 4.997 | 12.711 |  |
|  | **TT or TA** | 20 | 38.885 | 38.500 | 28.500 | 47.700 | 6.000 | 4.653 | 11.965 |  |
|  | **G present** | 58 | 39.321 | 39.700 | 22.300 | 46.600 | 5.700 | 4.445 | 11.306 | 0.5556** |
|  | **G absent** | 20 | 38.885 | 38.500 | 28.500 | 47.700 | 6.000 | 4.653 | 11.965 |  |
|  | **T or A present** | 53 | 39.151 | 39.000 | 22.300 | 47.700 | 6.900 | 4.829 | 12.334 | 0.9872** |
|  | **T or A absent** | 25 | 39.332 | 39.800 | 32.400 | 45.600 | 4.900 | 3.693 | 9.390 |  |
| **Ig** | **GG** | 23 | 0.235 | 0.120 | 0.002 | 0.700 | 0.350 | 0.226 | 95.976 | 0.2497* |
|  | **GT or GA** | 32 | 0.157 | 0.075 | 0.000 | 1.050 | 0.130 | 0.216 | 137.592 |  |
|  | **TT or TA** | 18 | 0.202 | 0.105 | 0.000 | 0.600 | 0.240 | 0.193 | 95.310 |  |
|  | **G present** | 55 | 0.190 | 0.100 | 0.000 | 1.050 | 0.260 | 0.221 | 116.764 | 0.6313** |
|  | **G absent** | 18 | 0.202 | 0.105 | 0.000 | 0.600 | 0.240 | 0.193 | 95.310 |  |
|  | **T or A present** | 50 | 0.173 | 0.085 | 0.000 | 1.050 | 0.190 | 0.207 | 119.512 | 0.2069** |
|  | **T or A absent** | 23 | 0.235 | 0.120 | 0.002 | 0.700 | 0.350 | 0.226 | 95.976 |  |
| **Neutro** | **GG** | 25 | 6.684 | 6.700 | 3.680 | 11.950 | 3.550 | 2.263 | 33.864 | 0.8588* |
|  | **GT or GA** | 33 | 6.754 | 6.050 | 2.220 | 16.570 | 3.460 | 3.187 | 47.190 |  |
|  | **TT or TA** | 20 | 7.051 | 6.410 | 3.660 | 15.600 | 3.370 | 2.808 | 39.829 |  |
|  | **G present** | 58 | 6.724 | 6.550 | 2.220 | 16.570 | 3.530 | 2.804 | 41.699 | 0.6554** |
|  | **G absent** | 20 | 7.051 | 6.410 | 3.660 | 15.600 | 3.370 | 2.808 | 39.829 |  |
|  | **T or A present** | 53 | 6.866 | 6.110 | 2.220 | 16.570 | 3.210 | 3.025 | 44.066 | 0.9190** |
|  | **T or A absent** | 25 | 6.684 | 6.700 | 3.680 | 11.950 | 3.550 | 2.263 | 33.864 |  |
| **Limfo** | **GG** | 25 | 1.845 | 1.740 | 0.750 | 3.640 | 0.880 | 0.773 | 41.869 | 0.6811* |
|  | **GT or GA** | 33 | 1.770 | 1.800 | 0.490 | 4.870 | 0.850 | 0.864 | 48.812 |  |
|  | **TT or TA** | 20 | 1.955 | 1.840 | 0.010 | 4.650 | 1.060 | 1.010 | 51.687 |  |
|  | **G present** | 58 | 1.803 | 1.770 | 0.490 | 4.870 | 0.960 | 0.820 | 45.474 | 0.4996** |
|  | **G absent** | 20 | 1.955 | 1.840 | 0.010 | 4.650 | 1.060 | 1.010 | 51.687 |  |
|  | **T or A present** | 53 | 1.840 | 1.820 | 0.010 | 4.870 | 1.070 | 0.917 | 49.833 | 0.8221** |
|  | **T or A absent** | 25 | 1.845 | 1.740 | 0.750 | 3.640 | 0.880 | 0.773 | 41.869 |  |
| **Mono** | **GG** | 25 | 0.722 | 0.620 | 0.000 | 1.430 | 0.440 | 0.307 | 42.583 | 0.4151* |
|  | **GT or GA** | 33 | 0.794 | 0.740 | 0.078 | 1.550 | 0.360 | 0.303 | 38.199 |  |
|  | **TT or TA** | 20 | 0.715 | 0.615 | 0.010 | 1.460 | 0.375 | 0.336 | 47.020 |  |
|  | **G present** | 58 | 0.763 | 0.710 | 0.000 | 1.550 | 0.370 | 0.304 | 39.916 | 0.4067** |
|  | **G absent** | 20 | 0.715 | 0.615 | 0.010 | 1.460 | 0.375 | 0.336 | 47.020 |  |
|  | **T or A present** | 53 | 0.764 | 0.710 | 0.010 | 1.550 | 0.410 | 0.315 | 41.259 | 0.5786# |
|  | **T or A absent** | 25 | 0.722 | 0.620 | 0.000 | 1.430 | 0.440 | 0.307 | 42.583 |  |
| **PLT** | **GG** | 25 | 279.200 | 237.000 | 151.000 | 536.000 | 97.000 | 94.787 | 33.950 | 0.7126* |
|  | **GT or GA** | 33 | 259.909 | 259.000 | 104.000 | 617.000 | 86.000 | 89.585 | 34.468 |  |
|  | **TT or TA** | 20 | 279.050 | 281.000 | 109.000 | 475.000 | 116.000 | 96.697 | 34.652 |  |
|  | **G present** | 58 | 268.224 | 258.500 | 104.000 | 617.000 | 95.000 | 91.550 | 34.132 | 0.5974** |
|  | **G absent** | 20 | 279.050 | 281.000 | 109.000 | 475.000 | 116.000 | 96.697 | 34.652 |  |
|  | **T or A present** | 53 | 267.132 | 263.000 | 104.000 | 617.000 | 93.000 | 91.885 | 34.397 | 0.7279** |
|  | **T or A absent** | 25 | 279.200 | 237.000 | 151.000 | 536.000 | 97.000 | 94.787 | 33.950 |  |
| **NLR** | **GG** | 25 | 4.304 | 3.327 | 1.224 | 10.693 | 2.356 | 2.525 | 58.666 | 0.8603* |
|  | **GT or GA** | 33 | 5.428 | 3.833 | 0.787 | 29.347 | 3.332 | 5.747 | 105.873 |  |
|  | **TT or TA** | 19 | 4.099 | 3.705 | 1.494 | 12.893 | 2.390 | 2.625 | 64.055 |  |
|  | **G present** | 58 | 4.944 | 3.549 | 0.787 | 29.347 | 2.990 | 4.641 | 93.888 | 0.5990** |
|  | **G absent** | 19 | 4.099 | 3.705 | 1.494 | 12.893 | 2.390 | 2.625 | 64.055 |  |
|  | **T or A present** | 52 | 4.942 | 3.769 | 0.787 | 29.347 | 3.020 | 4.855 | 98.238 | 0.9350** |
|  | **T or A absent** | 25 | 4.304 | 3.327 | 1.224 | 10.693 | 2.356 | 2.525 | 58.666 |  |
| **LMR** | **GG** | 24 | 2.704 | 2.786 | 0.608 | 5.870 | 1.501 | 1.146 | 42.373 | 0.5943* |
|  | **GT or GA** | 32 | 2.423 | 2.143 | 0.360 | 5.098 | 1.938 | 1.303 | 53.765 |  |
|  | **TT or TA** | 19 | 2.549 | 2.630 | 0.020 | 5.610 | 1.480 | 1.317 | 51.678 |  |
|  | **G present** | 56 | 2.544 | 2.425 | 0.360 | 5.870 | 1.918 | 1.235 | 48.556 | 0.9885# |
|  | **G absent** | 19 | 2.549 | 2.630 | 0.020 | 5.610 | 1.480 | 1.317 | 51.678 |  |
|  | **T or A present** | 51 | 2.470 | 2.440 | 0.020 | 5.610 | 1.700 | 1.296 | 52.486 | 0.4514# |
|  | **T or A absent** | 24 | 2.704 | 2.786 | 0.608 | 5.870 | 1.501 | 1.146 | 42.373 |  |
| **PLR** | **GG** | 25 | 203.787 | 152.000 | 62.000 | 976.000 | 75.448 | 176.047 | 86.388 | 0.6392* |
|  | **GT or GA** | 33 | 190.870 | 140.000 | 64.000 | 555.000 | 121.000 | 117.413 | 61.515 |  |
|  | **TT or TA** | 19 | 151.913 | 141.398 | 47.000 | 323.864 | 70.000 | 65.480 | 43.104 |  |
|  | **G present** | 58 | 196.438 | 148.589 | 62.000 | 976.000 | 119.000 | 144.328 | 73.473 | 0.4048** |
|  | **G absent** | 19 | 151.913 | 141.398 | 47.000 | 323.864 | 70.000 | 65.480 | 43.104 |  |
|  | **T or A present** | 52 | 176.636 | 140.699 | 47.000 | 555.000 | 100.000 | 102.577 | 58.073 | 0.4661* |
|  | **T or A absent** | 25 | 203.787 | 152.000 | 62.000 | 976.000 | 75.448 | 176.047 | 86.388 |  |

* Kruskal-Wallis ANOVA; ** Mann-Whitney’s U test; # Student’s t test

**Supplement table B4**. Characterization of age at the lung cancer disease onset and blood morphology indices according to C3435T *ABCB1* genotype and allele status

|  | **3435** | **N** | **Mean** | **Median** | **Min.** | **Max.** | **IQR** | **SD** | **CV** | **P value*** |
| --- | --- | --- | --- | --- | --- | --- | --- | --- | --- | --- |
| **Age** | **CC** | 11 | 69.000 | 71.000 | 56.000 | 76.000 | 8.000 | 6.083 | 8.816 | 0.5642* |
|  | **CT** | 41 | 67.390 | 68.000 | 53.000 | 82.000 | 7.000 | 7.078 | 10.503 |  |
|  | **TT** | 28 | 66.571 | 68.000 | 32.000 | 80.000 | 10.500 | 10.046 | 15.090 |  |
|  | C present | 52 | 67.731 | 68.000 | 53.000 | 82.000 | 7.500 | 6.855 | 10.120 | 0.8205** |
|  | C absent | 28 | 66.571 | 68.000 | 32.000 | 80.000 | 10.500 | 10.046 | 15.090 |  |
|  | T present | 69 | 67.058 | 68.000 | 32.000 | 82.000 | 8.000 | 8.349 | 12.450 | 0.3565** |
|  | T absent | 11 | 69.000 | 71.000 | 56.000 | 76.000 | 8.000 | 6.083 | 8.816 |  |
| **WBC** | **CC** | 11 | 8.988 | 9.070 | 6.020 | 11.280 | 1.620 | 1.498 | 16.664 | 0.2257* |
|  | **CT** | 41 | 9.130 | 8.480 | 4.540 | 16.260 | 4.000 | 2.957 | 32.388 |  |
|  | **TT** | 28 | 10.415 | 10.270 | 5.500 | 19.390 | 3.480 | 3.354 | 32.208 |  |
|  | C present | 52 | 9.100 | 8.845 | 4.540 | 16.260 | 3.695 | 2.702 | 29.693 | 0.0882** |
|  | C absent | 28 | 10.415 | 10.270 | 5.500 | 19.390 | 3.480 | 3.354 | 32.208 |  |
|  | T present | 69 | 9.652 | 9.070 | 4.540 | 19.390 | 3.710 | 3.165 | 32.789 | 0.7853** |
|  | T absent | 11 | 8.988 | 9.070 | 6.020 | 11.280 | 1.620 | 1.498 | 16.664 |  |
| **RBC** | **CC** | 11 | 4.407 | 4.460 | 3.460 | 5.000 | 0.610 | 0.456 | 10.347 | 0.9448* |
|  | **CT** | 41 | 4.430 | 4.570 | 2.400 | 5.310 | 0.710 | 0.561 | 12.665 |  |
|  | **TT** | 28 | 4.401 | 4.340 | 2.970 | 5.290 | 0.945 | 0.551 | 12.519 |  |
|  | C present | 52 | 4.426 | 4.555 | 2.400 | 5.310 | 0.710 | 0.536 | 12.123 | 0.8009** |
|  | C absent | 28 | 4.401 | 4.340 | 2.970 | 5.290 | 0.945 | 0.551 | 12.519 |  |
|  | T present | 69 | 4.419 | 4.450 | 2.400 | 5.310 | 0.810 | 0.553 | 12.518 | 0.8999** |
|  | T absent | 11 | 4.407 | 4.460 | 3.460 | 5.000 | 0.610 | 0.456 | 10.347 |  |
| **HTC** | **CC** | 11 | 39.609 | 40.100 | 32.400 | 45.300 | 4.800 | 3.535 | 8.926 | 0.9361* |
|  | **CT** | 41 | 39.198 | 39.500 | 22.300 | 47.700 | 5.700 | 4.574 | 11.670 |  |
|  | **TT** | 28 | 39.064 | 39.100 | 28.500 | 46.500 | 8.050 | 4.626 | 11.841 |  |
|  | C present | 52 | 39.285 | 39.650 | 22.300 | 47.700 | 5.300 | 4.346 | 11.064 | 0.7699** |
|  | C absent | 28 | 39.064 | 39.100 | 28.500 | 46.500 | 8.050 | 4.626 | 11.841 |  |
|  | T present | 69 | 39.143 | 39.200 | 22.300 | 47.700 | 6.300 | 4.562 | 11.654 | 0.7799** |
|  | T absent | 11 | 39.609 | 40.100 | 32.400 | 45.300 | 4.800 | 3.535 | 8.926 |  |
| **Ig** | **CC** | 11 | 0.237 | 0.110 | 0.002 | 0.700 | 0.460 | 0.252 | 106.657 | 0.8980* |
|  | **CT** | 37 | 0.180 | 0.100 | 0 | 1.050 | 0.150 | 0.216 | 119.771 |  |
|  | **TT** | 26 | 0.187 | 0.085 | 0 | 0.600 | 0.260 | 0.196 | 105.104 |  |
|  | C present | 48 | 0.193 | 0.100 | 0 | 1.050 | 0.250 | 0.223 | 115.558 | 0.6877** |
|  | C absent | 26 | 0.187 | 0.085 | 0 | 0.600 | 0.260 | 0.196 | 105.104 |  |
|  | T present | 63 | 0.183 | 0.100 | 0.000 | 1.050 | 0.250 | 0.206 | 112.824 | 0.7439** |
|  | T absent | 11 | 0.237 | 0.110 | 0.002 | 0.700 | 0.460 | 0.252 | 106.657 |  |
| **Neutro** | **CC** | 11 | 6.601 | 6.700 | 3.900 | 8.960 | 2.510 | 1.613 | 24.440 | 0.2564* |
|  | **CT** | 41 | 6.273 | 5.460 | 2.220 | 14.380 | 3.510 | 2.687 | 42.836 |  |
|  | **TT** | 28 | 7.454 | 6.715 | 3.660 | 16.570 | 3.695 | 3.217 | 43.162 |  |
|  | C present | 52 | 6.342 | 5.825 | 2.220 | 14.380 | 3.530 | 2.488 | 39.233 | 0.1535** |
|  | C absent | 28 | 7.454 | 6.715 | 3.660 | 16.570 | 3.695 | 3.217 | 43.162 |  |
|  | T present | 69 | 6.752 | 6.050 | 2.220 | 16.570 | 3.490 | 2.949 | 43.680 | 0.7216** |
|  | T absent | 11 | 6.601 | 6.700 | 3.900 | 8.960 | 2.510 | 1.613 | 24.440 |  |
| **Limfo** | **CC** | 11 | 1.577 | 1.550 | 0.750 | 2.420 | 1.260 | 0.587 | 37.229 | 0.6412 |
|  | **CT** | 41 | 1.867 | 1.910 | 0.010 | 4.870 | 0.960 | 0.894 | 47.910 |  |
|  | **TT** | 28 | 1.927 | 1.780 | 0.560 | 4.650 | 1.060 | 0.897 | 46.555 |  |
|  | C present | 52 | 1.806 | 1.810 | 0.010 | 4.870 | 0.910 | 0.842 | 46.643 | 0.5756** |
|  | C absent | 28 | 1.927 | 1.780 | 0.560 | 4.650 | 1.060 | 0.897 | 46.555 |  |
|  | T present | 69 | 1.891 | 1.860 | 0.010 | 4.870 | 1.000 | 0.889 | 47.026 | 0.3788** |
|  | T absent | 11 | 1.577 | 1.550 | 0.750 | 2.420 | 1.260 | 0.587 | 37.229 |  |
| **Mono** | **CC** | 11 | 0.645 | 0.610 | 0.000 | 1.430 | 0.180 | 0.347 | 53.907 | 0.2774* |
|  | **CT** | 41 | 0.729 | 0.620 | 0.078 | 1.550 | 0.410 | 0.294 | 40.294 |  |
|  | **TT** | 28 | 0.803 | 0.735 | 0.010 | 1.460 | 0.540 | 0.320 | 39.875 |  |
|  | C present | 52 | 0.711 | 0.620 | 0 | 1.550 | 0.380 | 0.304 | 42.781 | 0.1478** |
|  | C absent | 28 | 0.802 | 0.735 | 0.010 | 1.460 | 0.540 | 0.320 | 39.875 |  |
|  | T present | 69 | 0.759 | 0.710 | 0.010 | 1.550 | 0.420 | 0.304 | 40.133 | 0.2883** |
|  | T absent | 11 | 0.645 | 0.610 | 0 | 1.430 | 0.180 | 0.347 | 53.907 |  |
| **PLT** | **CC** | 11 | 238.182 | 235.000 | 151.000 | 301.000 | 67.000 | 47.573 | 19.973 | 0.4375* |
|  | **CT** | 41 | 274.220 | 267.000 | 104.000 | 536.000 | 114.000 | 90.9378 | 33.162 |  |
|  | **TT** | 28 | 277.750 | 256.000 | 109.000 | 617.000 | 88.000 | 108.270 | 38.981 |  |
|  | C present | 52 | 266.596 | 264.000 | 104.000 | 536.000 | 101.500 | 84.561 | 31.719 | 0.9357** |
|  | C absent | 28 | 277.750 | 256.000 | 109.000 | 617.000 | 88.000 | 108.270 | 38.981 |  |
|  | T present | 69 | 275.652 | 265.000 | 104.000 | 617.000 | 108.000 | 97.581 | 35.400 | 0.2323** |
|  | T absent | 11 | 238.182 | 235.000 | 151.000 | 301.000 | 67.000 | 47.573 | 19.973 |  |
| **NLR** | **CC** | 11 | 5.221 | 3.656 | 1.981 | 10.693 | 6.726 | 3.344 | 64.058 | 0.5189* |
|  | **CT** | 40 | 4.221 | 3.267 | 0.787 | 29.347 | 2.454 | 4.433 | 105.028 |  |
|  | **TT** | 28 | 5.069 | 3.769 | 1.224 | 20.393 | 2.993 | 4.239 | 83.625 |  |
|  | C present | 51 | 4.436 | 3.327 | 0.787 | 29.347 | 2.854 | 4.211 | 94.932 | 0.6631** |
|  | C absent | 28 | 5.069 | 3.769 | 1.224 | 20.393 | 2.993 | 4.239 | 83.625 |  |
|  | T present | 68 | 4.570 | 3.498 | 0.787 | 29.347 | 2.806 | 4.342 | 95.020 | 0.3838** |
|  | T absent | 11 | 5.221 | 3.656 | 1.981 | 10.693 | 6.726 | 3.344 | 64.058 |  |
| **LMR** | **CC** | 10 | 2.575 | 2.573 | 0.608 | 4.033 | 1.350 | 1.012 | 39.291 | 0.2976*** |
|  | **CT** | 40 | 2.803 | 2.780 | 0.020 | 5.870 | 2.498 | 1.467 | 52.340 |  |
|  | **TT** | 27 | 2.306 | 2.354 | 0.680 | 4.780 | 1.530 | 1.015 | 43.995 |  |
|  | C present | 50 | 2.758 | 2.780 | 0.020 | 5.870 | 1.871 | 1.382 | 50.118 | 0.1397# |
|  | C absent | 27 | 2.306 | 2.354 | 0.680 | 4.780 | 1.530 | 1.015 | 43.995 |  |
|  | T present | 67 | 2.603 | 2.530 | 0.020 | 5.870 | 1.877 | 1.318 | 50.649 | 0.9784# |
|  | T absent | 10 | 2.575 | 2.573 | 0.608 | 4.033 | 1.350 | 1.012 | 39.291 |  |
| **PLR** | **CC** | 11 | 197.499 | 174.074 | 99.537 | 340.000 | 159.000 | 82.612 | 41.829 | 0.4814* |
|  | **CT** | 40 | 199.293 | 145.500 | 61.000 | 976.000 | 122.405 | 163.194 | 81.886 |  |
|  | **TT** | 28 | 165.590 | 146.699 | 47.000 | 392.000 | 97.500 | 83.033 | 50.144 |  |
|  | C present | 51 | 198.906 | 149.677 | 61.000 | 976.000 | 123.810 | 148.791 | 74.804 | 0.4826** |
|  | C absent | 28 | 165.590 | 146.699 | 47.000 | 392.000 | 97.500 | 83.033 | 50.144 |  |
|  | T present | 68 | 185.415 | 145.500 | 47.000 | 976.000 | 98.000 | 136.235 | 73.476 | 0.2543** |
|  | T absent | 11 | 197.499 | 174.074 | 99.537 | 340.000 | 159.000 | 82.612 | 41.829 |  |

* Kruskal-Wallis ANOVA; ** Mann-Whitney U test; ***ANOVA; # Student t test
