# Supplementary figures and images for: The Interaction between Four Polymorphisms and Haplotype of ABCB1, the Risk of Non-Small Cell Lung Cancer, and the Disease Phenotype
Source: J Oncol. 2023 Jan 24;2023:7925378. doi: 10.1155/2023/7925378 (PMC9902128; doi:10.1155/2023/7925378)

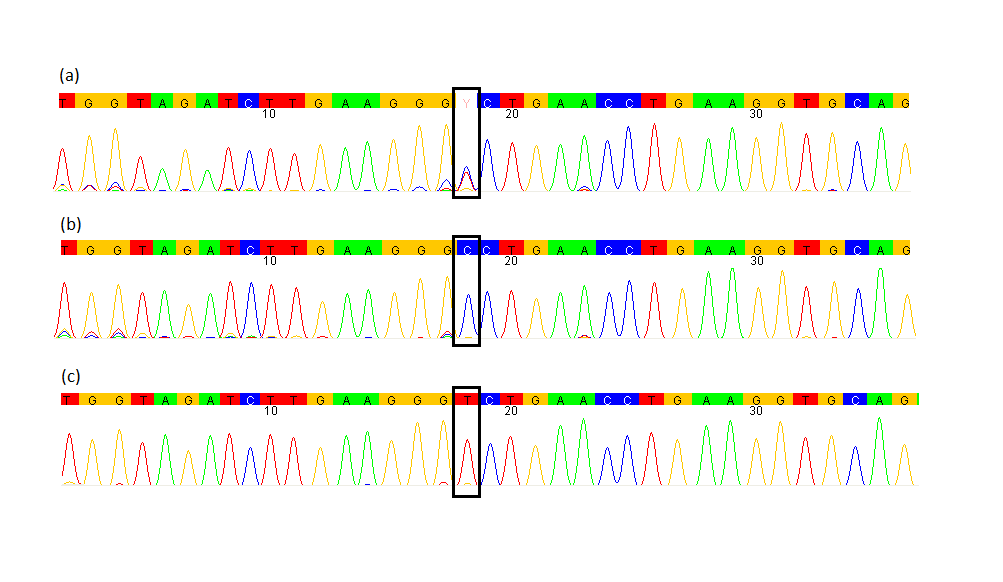

Supplement: Supplementary Materials — Supplement Figure 1: examples of a separation of ABCB1 fragment. Sanger sequencing chromatograms showing polymorphism C1236T in (a) CT heterozygous, (b) CC homozygous, and (c) TT homozygous. Supplement Figure 2: examples of a separation of ABCB1 fragment. Sanger sequencing chromatograms showing polymorphism G2677T/A in (a) TT homozygous, (b) GG homozygous, (c) GT heterozygous, (d) TA heterozygous, and (e) GA heterozygous. Supplement Table A1: genotype frequencies of ABCB1 gene T-129C polymorphism according to clinicopathological parameters. Supplement Table A2: genotype and allele frequencies of ABCB1 gene C1236T polymorphism according to clinicopathological parameters. Supplement Table A3: genotype and allele frequencies of ABCB1 gene G2677T/A polymorphism according to clinicopathological parameters. Supplement Table A4: genotype and allele frequencies of ABCB1 gene C3435T polymorphism according to clinicopathological parameters. Supplement Table B1: characterization of age at lung cancer disease onset and blood morphology indices according to T-129C ABCB1 genotype. Supplement Table B2: characterization of age at lung cancer disease onset and blood morphology indices according to C1236T ABCB1 genotype and allele status. Supplement Table B3: characterization of age at lung cancer disease onset and blood morphology indices according to G2677T/A ABCB1 genotype and allele status. Supplement Table B4: characterization of age at lung cancer disease onset and blood morphology indices according to C3435T ABCB1 genotype and allele status. [file 7925378.f1.zip › Supplement Figure 1 (1).png]

(a)

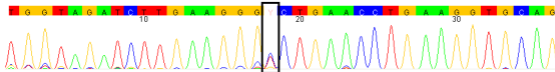

(b)

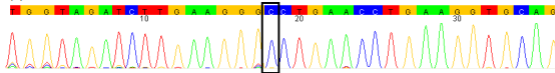

(c)

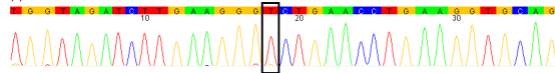

Supplement: Supplementary Materials — Supplement Figure 1: examples of a separation of ABCB1 fragment. Sanger sequencing chromatograms showing polymorphism C1236T in (a) CT heterozygous, (b) CC homozygous, and (c) TT homozygous. Supplement Figure 2: examples of a separation of ABCB1 fragment. Sanger sequencing chromatograms showing polymorphism G2677T/A in (a) TT homozygous, (b) GG homozygous, (c) GT heterozygous, (d) TA heterozygous, and (e) GA heterozygous. Supplement Table A1: genotype frequencies of ABCB1 gene T-129C polymorphism according to clinicopathological parameters. Supplement Table A2: genotype and allele frequencies of ABCB1 gene C1236T polymorphism according to clinicopathological parameters. Supplement Table A3: genotype and allele frequencies of ABCB1 gene G2677T/A polymorphism according to clinicopathological parameters. Supplement Table A4: genotype and allele frequencies of ABCB1 gene C3435T polymorphism according to clinicopathological parameters. Supplement Table B1: characterization of age at lung cancer disease onset and blood morphology indices according to T-129C ABCB1 genotype. Supplement Table B2: characterization of age at lung cancer disease onset and blood morphology indices according to C1236T ABCB1 genotype and allele status. Supplement Table B3: characterization of age at lung cancer disease onset and blood morphology indices according to G2677T/A ABCB1 genotype and allele status. Supplement Table B4: characterization of age at lung cancer disease onset and blood morphology indices according to C3435T ABCB1 genotype and allele status. [file 7925378.f1.zip › Supplement Figure 1.pdf]

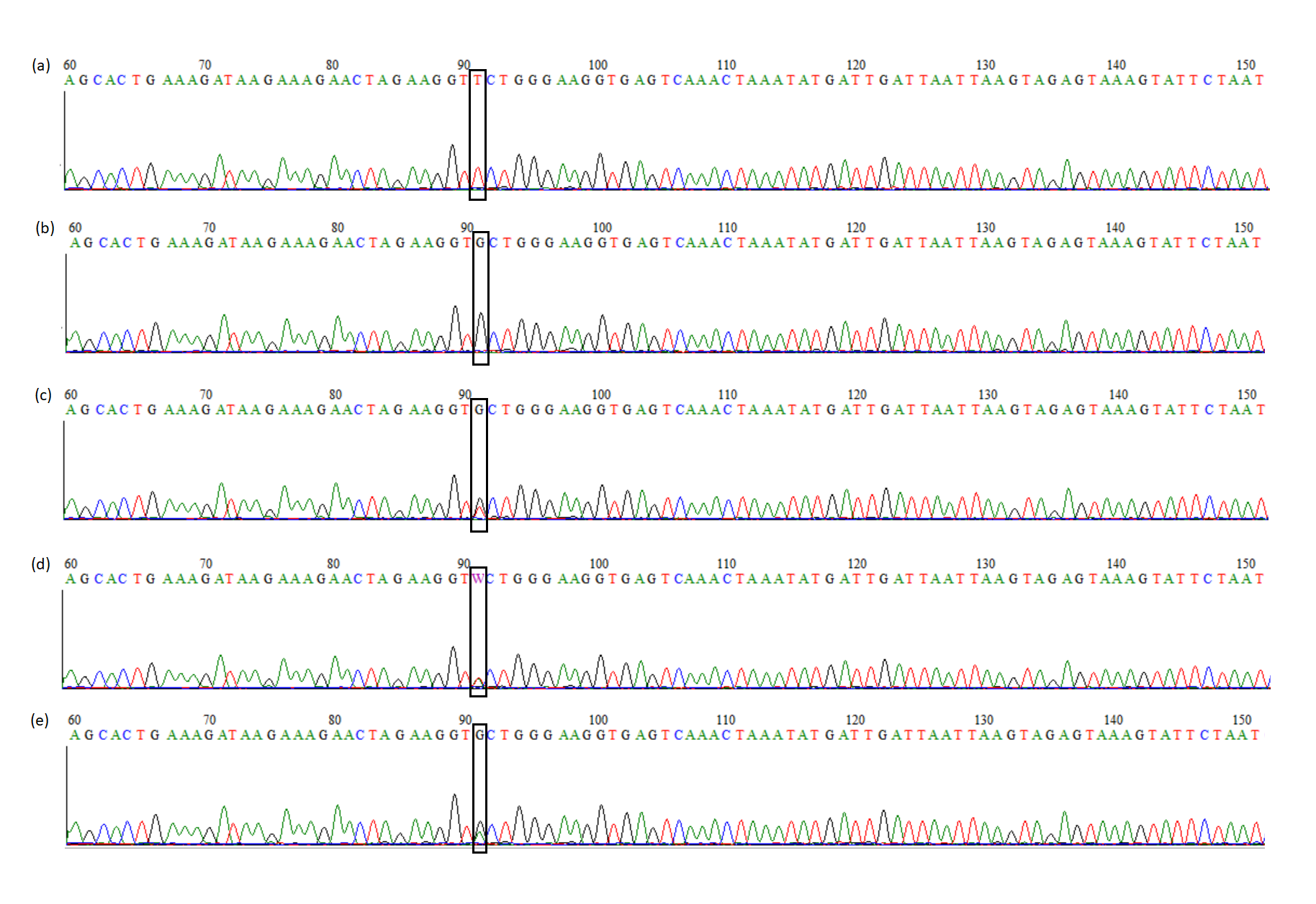

Supplement: Supplementary Materials — Supplement Figure 1: examples of a separation of ABCB1 fragment. Sanger sequencing chromatograms showing polymorphism C1236T in (a) CT heterozygous, (b) CC homozygous, and (c) TT homozygous. Supplement Figure 2: examples of a separation of ABCB1 fragment. Sanger sequencing chromatograms showing polymorphism G2677T/A in (a) TT homozygous, (b) GG homozygous, (c) GT heterozygous, (d) TA heterozygous, and (e) GA heterozygous. Supplement Table A1: genotype frequencies of ABCB1 gene T-129C polymorphism according to clinicopathological parameters. Supplement Table A2: genotype and allele frequencies of ABCB1 gene C1236T polymorphism according to clinicopathological parameters. Supplement Table A3: genotype and allele frequencies of ABCB1 gene G2677T/A polymorphism according to clinicopathological parameters. Supplement Table A4: genotype and allele frequencies of ABCB1 gene C3435T polymorphism according to clinicopathological parameters. Supplement Table B1: characterization of age at lung cancer disease onset and blood morphology indices according to T-129C ABCB1 genotype. Supplement Table B2: characterization of age at lung cancer disease onset and blood morphology indices according to C1236T ABCB1 genotype and allele status. Supplement Table B3: characterization of age at lung cancer disease onset and blood morphology indices according to G2677T/A ABCB1 genotype and allele status. Supplement Table B4: characterization of age at lung cancer disease onset and blood morphology indices according to C3435T ABCB1 genotype and allele status. [file 7925378.f1.zip › Supplement Figure 2 (1).png]

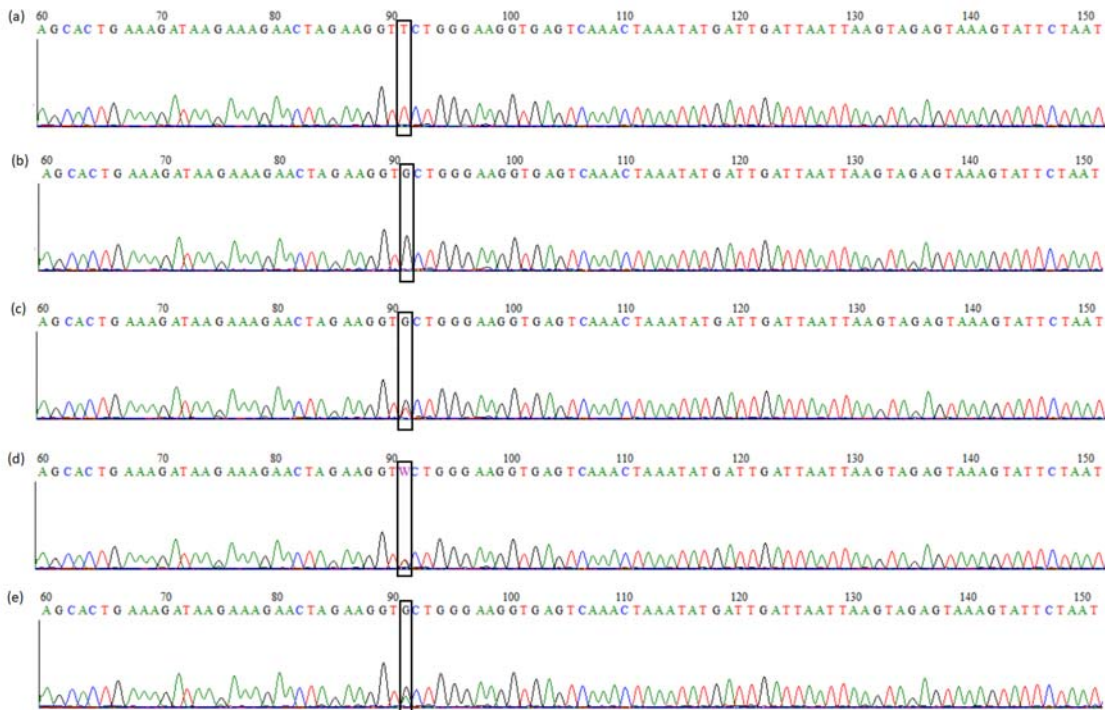

Supplement: Supplementary Materials — Supplement Figure 1: examples of a separation of ABCB1 fragment. Sanger sequencing chromatograms showing polymorphism C1236T in (a) CT heterozygous, (b) CC homozygous, and (c) TT homozygous. Supplement Figure 2: examples of a separation of ABCB1 fragment. Sanger sequencing chromatograms showing polymorphism G2677T/A in (a) TT homozygous, (b) GG homozygous, (c) GT heterozygous, (d) TA heterozygous, and (e) GA heterozygous. Supplement Table A1: genotype frequencies of ABCB1 gene T-129C polymorphism according to clinicopathological parameters. Supplement Table A2: genotype and allele frequencies of ABCB1 gene C1236T polymorphism according to clinicopathological parameters. Supplement Table A3: genotype and allele frequencies of ABCB1 gene G2677T/A polymorphism according to clinicopathological parameters. Supplement Table A4: genotype and allele frequencies of ABCB1 gene C3435T polymorphism according to clinicopathological parameters. Supplement Table B1: characterization of age at lung cancer disease onset and blood morphology indices according to T-129C ABCB1 genotype. Supplement Table B2: characterization of age at lung cancer disease onset and blood morphology indices according to C1236T ABCB1 genotype and allele status. Supplement Table B3: characterization of age at lung cancer disease onset and blood morphology indices according to G2677T/A ABCB1 genotype and allele status. Supplement Table B4: characterization of age at lung cancer disease onset and blood morphology indices according to C3435T ABCB1 genotype and allele status. [file 7925378.f1.zip › Supplement Figure 2.pdf]
